# Arterial Blood Gas Analysis of Patients with Tramadol-induced Seizure; a Cross Sectional Study 

**Published:** 2020-03-01

**Authors:** Bita Dadpour, Anahita Alizadeh, Maryam Vahabzadeh, Seyed Reza Mousavi, Mohammad Moshiri, Zahra Ataee, Babak Mostafazadeh

**Affiliations:** 1 Medical Toxicology Research Center, Mashhad University of Medical Sciences, Mashhad, Iran.; 2 Toxicological Research Center, Shahid Beheshti University of Medical Sciences, Tehran, Iran.; 3 Department of Forensic Medicine and Toxicology, Shahid Beheshti University of Medical Sciences, Tehran, Iran.

**Keywords:** Tramadol, blood gas analysis, seizures, acidosis, respiratory, hypercapnia

## Abstract

**Introduction:**

Tramadol is an active analgesic drug that is commonly used to treat moderate to severe pain. The present study aimed to assess the arterial blood gas (ABG) analysis of patients with tramadol-induced seizure (TIS).

**Methods:**

This prospective cross-sectional study was performed on 50 TIS cases that were referred to emergency department within a maximum of one hour after their last episode of seizure. The results of ABG analysis on admission were collected and their association with dosage and time interval between ingestion and admission was assessed.

**Results:**

50 cases with the mean age of 35.10 ± 9.62 years were studied (80.0% male). The mean dosage of ingestion was 1122.00 ± 613.88 (400 to 3000) mg and the mean time interval between ingestion and admission was 7.16 ± 2.18 hours. ABG analysis on admission showed that 49 (98.0%) patients had pH < 7.35 and PaCO2 > 45 mmHg (respiratory acidosis). There was a significant association between ingestion to admission time interval and both PaCO_2_ (r = -0.330, p = 0.019), and PaO_2_ (r = 0.303, p = 0.032). The dose of ingestion was negatively associated with respiratory rate (r = -0.556, p = 0.001), arterial pH (r = -0.676, p = 0.001), and PaO_2_ (r = -0.514, p = 0.001), but was positively associated with PaCO_2_ (r = 0.461, p = 0.001). Higher doses of tramadol led to more severe hypercapnia and need for intubation (OR = 1.12, 95% CI: 0.88 – 1.26; p = 0.045). 5 (10.0%) cases needed mechanical ventilation. All patients improved after supportive care with no in-hospital death.

**Conclusion:**

Based on the findings, 98% of TIS cases had respiratory acidosis. Higher doses of ingested drug and longer time interval between ingestion and admission were associated with severity of ABG disturbances.

## Introduction

Tramadol is an active analgesic drug, which is commonly used to treat moderate to severe pain with different sources. According to the literature, this analgesic agent is one of the most prescribed opioids worldwide (1, 2). The mechanism of action of the drug is stimulation of μ-opioid receptor as well as inhibition of serotonin and noradrenaline reuptake (3). However, the analgesic effect of the drug is mainly dependent on its non-opioid properties and through activation of central monoaminergic pathways (4). Due to its high efficacy, especially in pain relief, the misuse of tramadol has been reported in almost all clinical settings in the world; leading to potential complications such as seizure, which has been reported in 15% to 35% of patients (5, 6). 

The exact mechanisms of tramadol-induced seizure (TIS) remains unexplained; however, it seems that its inhibitory effects on gamma-aminobutyric acid (GABA) receptors along with its opioid receptor agonist activity play pivotal roles (7). TIS may appear by consuming recommended doses (8). Moreover, the risk of seizure occurring may also synergistically increase by simultaneous use of other drugs such as phenothiazines, tricyclic antidepressants, and selective serotonin reuptake inhibitors (9, 10). 

There are two important points about TIS. First, the minimum stimulant dose of drug that causes seizures and also its blood concentrations remain unknown. In addition, there are some evidence for effects of tramadol on arterial blood gas (ABG) disturbances, especially rise in carbon dioxide pressure (PCO2), which leads to respiratory depression (11). In this regard, we hypothesize that occurrence of seizure following tramadol use may be related to increased blood PCO2. The present study aimed to assess arterial blood gas (ABG) analysis of patients with tramadol-induced seizure (TIS).  

## Methods

***Study design and setting***

This prospective cross-sectional study was performed on 50 cases that were referred to emergency department of Payambaran Hospital, Tehran, Iran, within a maximum of one hour after TIS (patients with a history of tramadol ingestion followed by generalized tonic-clonic seizure), from July to December 2019. The results of ABG analysis on admission were collected and their association with dosage and time interval between ingestion and admission was assessed. The protocol of study was approved by Ethics Committee of Shahid Beheshti University of Medical Sciences, Tehran, Iran (Ethics Code: IR.SBMU.RETECH.REC.1398.355).

***Participants***

Patients with a history of tramadol use with subsequent generalized seizure (diagnosed as tramadol-induced seizure) that were referred to emergency department of our hospital within a maximum of one hour after last episode of seizure were included in this study. Patients with history of head trauma, multi drug ingestion, positive amphetamine test, history of methamphetamine, morphine, or methadone abuse, the use of psychological medications such as tricyclic antidepressants, phenothiazine, or selective serotonin reuptake inhibitors, presenting to the hospital more than one hour after the last seizure episode, or any other reason for the seizure, were excluded.

***Data gathering***

On admission, the patients’ baseline characteristics (gender, age, time and dose of taking medication, history of seizures before hospitalization), the level of consciousness (according to the Richmond Agitation-Sedation Scale), vital signs (blood pressure, respiratory rate, pulse rate, temperature), need for mechanical ventilation, and at the time of discharge, duration of hospital stay were collected using a predesigned checklist. Also, the results of ABG analysis were collected on admission and then 12 hours after the initial assessment. An expert toxicologist was responsible for data gathering.

***Statistical analysis***

The results were presented as mean ± standard deviation (SD) for quantitative variables and were summarized by absolute frequencies and percentages for categorical variables. Normality of data was analyzed using the Kolmogorov-Smirnoff test. Categorical variables were compared using chi-square test or Fisher's exact test. Quantitative variables were also compared using t test, or Mann-Whitney U test. The association between the quantitative variables was tested via Pearson’s correlation test. To assess the relationship of time and dose of tramadol used with the change in ABG parameters with the presence of other variables as the confounders, the multivariable regression model was employed. For the statistical analysis, the statistical software SPSS version 16.0 for windows (SPSS Inc., Chicago, IL) was used. P values of 0.05 or less were considered statistically significant. 

## Results

***Baseline characteristics of studied cases***

50 tramadol-induced seizure cases with the mean age of 35.10 ± 9.62 (range: 25-45) years were studied (80.0% male). The mean dosage of tramadol used was 1122.00 ± 613.88 (400 to 3000) mg that led to one, two and three episodes of Pre-hospital seizures in 72.0%, 26.0%, and 2.0% of patients, respectively. The patients’ characteristics on admission are summarized in table 1. The mean time interval between use and admission was 7.16 ± 2.18 hours.

**Table 1:** Baseline characteristics of the study population

Variable	Value
Gender	
Male	41 (82.0)
Female	9 (18.0)
Age (year)	
Mean ± SD	35.10 ± 9.62
Time between tramadol use and admission (hours)	
< 3	9(18.0)
3 - 6	24(48.0)
≥ 6	17(34.0)
Dose of drug used (mg)	
< 500	8(16.0)
500 - 1000	29(58.0)
≥ 1000	13(26.0)
Medical history	
Chronic use of tramadol	30 (60.0)
Previous seizure	9 (18.0)
Epilepsy	1 (2.0)
Pre-hospital seizure frequency	
One time	36 (72.0)
Two times	13 (26.0)
Three times	1 (2.0)
In-hospital seizure	
Yes	9 (18.0)
No	41 (82.0)
Need for intubation	
Yes	5 (10.0)
No	45 (90.0)
Level of consciousness (RASS score)	
-1	2 (4.0)
-2	33 (66.0)
-3	14 (28.0)
-4	1 (2.0)

Data are presented as mean ± standard deviation or frequency (%). RASS: Richmond Agitation-Sedation Scale.

**Table 2:** Vital signs and blood gas analysis changes during the 12-hour monitoring

**Parameter **	**On admission **	**After 12 hours **	**P value**
Blood pressure	108.21 ± 20.14	111.90 ± 10.72	0.159
Heart rate	104.46 ± 9.09	94.43 ± 7.38	< 0.001
Respiratory rate	9.88 ± 1.45	12.61 ± 0.81	< 0.001
Body temperature	37.11 ± 0.26	36.98 ± 0.12	< 0.001
PH	7.28 ± 0.03	7.33 ± 0.02	< 0.001
HCO3 level	17.58 ± 1.29	20.06 ± 1.20	< 0.001
PCO_2_	53.80 ± 5.94	46.76 ± 2.56	< 0.001
PaO_2_	88.24 ± 2.98	95.68 ± 1.20	< 0.001

Data are presented as mean ± standard deviation.

***Blood gas analysis***

Arterial blood gas analysis on admission showed that 49 (98.0%) patients had pH < 7.35 and PCO2 > 45 mmHg (respiratory acidosis). There was a significant association between tramadol use to seizure time interval and blood pressure (r = 0.308, p = 0.030), heart rate (r = -0.441, p = 0.001), body temperature (r = -0.281, p = 0.048), PCO2 (r = -0.330, p = 0.019), and PO2 (r = 0.303, p = 0.032). The dose of medication used was negatively associated with blood pressure (r = -0.351, p = 0.030), respiratory rate (r = -0.556, p = 0.001), arterial pH (r = -0.676, p = 0.001), and PaO2 (r = -0.514, p = 0.001), but was positively associated with PCO2 (r = 0.461, p = 0.001). Higher doses of tramadol led to more severe hypercapnia and need for tracheal intubation (OR = 1.12, 95% CI: 0.88 – 1.26; p = 0.045). 

***Outcomes***

In most patients, the blood gas indices had significantly improved within 12 hours via supportive approaches (table 2). 5 (10.0%) cases needed mechanical ventilation. All patients improved after supportive care with no in-hospital death. The mean length of hospital stay was 2.04 ± 0.92 days (ranged 1 to 5 days). Higher dose of tramadol used was closely associated with longer hospital stay (beta = 0.683, p = 0.001).    

## Discussion

Based on the findings of the present study, higher doses of tramadol were associated with worse in-hospital outcome and led to more severe acid-base disturbances, which manifested as respiratory acidosis and hypercapnea. On the other hand, higher doses of tramadol use before admission can predict severe in-hospital complication and therefore, more severe blood gas disturbances in affected patients. 

Review of the literature indicated the risk for generalized seizure in up to 41% of tramadol users. However, the studies had also revealed that the likelihood of tramadol-induced seizure depends on various factors such as the definition and classification of seizures or simultaneous use of other analgesics such as codeine (12). Furthermore, these studies demonstrate an increased risk of seizure only at the highest level of tramadol exposure. However, some other studies showed the risk of seizure with even moderate doses of drug. In other words, the association between time and dose of tramadol consumption and the risk of seizure occurrence remains uncertain. 

As clearly determined in the present study, first, higher doses of tramadol was associated with worse in-hospital outcome such as more respiratory depression, more need for tracheal intubation and also longer hospital stay. In other words, consuming higher doses of tramadol led to more severe acid-base disturbances manifested as respiratory acidosis and hypercapnea that might lead to worse outcome. Thus, higher PaCO2 may be predictable in patients receiving high pre-hospital tramadol dosages. 

Respiratory effects of tramadol have been previously described along with other potential side effects, especially acid-base disturbances. In a study by Tantry et al. in 2011, a patient scheduled for thigh reduction-plasty was candidate for pain relief using tramadol with a moderate dose (200mg), which led to severe respiratory acidosis leading to emergency intubation and mechanical ventilation. In another experiment by Ismail et al. (13), about one-third of patients who had received tramadol with a 1600 mg dose suffered from respiratory acidosis with considerably raised PCO2. Also, as clearly shown by Rahimi et al. (14), the mean ingested dose of 1971.2 mg (range: 100-20000 mg) tramadol led to occurrence of seizure in 47.91% and pure acute respiratory acidosis  in almost all tramadol-intoxicated patients. Also, similar to our study, they indicated significant differences between cases with seizure and cases without seizure according to time interval between tramadol ingestion and hospital admission as well as ingested dose of drug. 

Therefore, summing our findings and the results of previous studies shows that in patients suffering from TIS, higher doses of tramadol and also longer time interval between tramadol ingestion and hospital admission may result in more severe acid-base disturbances such as respiratory acidosis. 

The association between the occurrence of seizure and the change in acid-base balance in TIS has not been previously examined. 

## Discussion

Respiratory changes with hypercapnia and hypoxemia, which can occur with seizures, have been exclusively studied in previous experiments. However, in our study and due to the lack of designing a case-control study with both seizure and non-seizure subgroups, assessing the relationship between the likelihood of seizure and respiratory acidosis following the use of high dose of tramadol was impossible, which should be considered as a major target in future studies. 

## Conclusion:

Based on the findings, 98% of TIS cases had respiratory acidosis. Higher doses of ingested drug and also longer time interval between ingestion and admission were associated with higher severity of ABG disturbances. 
